# One-Pot Biocatalytic Synthesis of *rac*-Syringaresinol from a Lignin-Derived Phenol

**DOI:** 10.1021/acscatal.3c04399

**Published:** 2023-10-31

**Authors:** Yiming Guo, Laura Alvigini, Mohammad Saifuddin, Ben Ashley, Milos Trajkovic, Lur Alonso-Cotchico, Andrea Mattevi, Marco W. Fraaije

**Affiliations:** † Molecular Enzymology Group, University of Groningen, Nijenborgh 4, Groningen, AG 9747, The Netherlands; ‡ Department of Biology and Biotechnology “Lazzaro Spallanzani”, University of Pavia, Via Ferrata 9, Pavia 27100, Italy; § Zymvol Biomodeling S.L., Carrer Roc Boronat, 117, Barcelona 08010, Spain

**Keywords:** syringaresinol, enzyme cascade, enzyme engineering, thermostability, one-pot synthesis

## Abstract

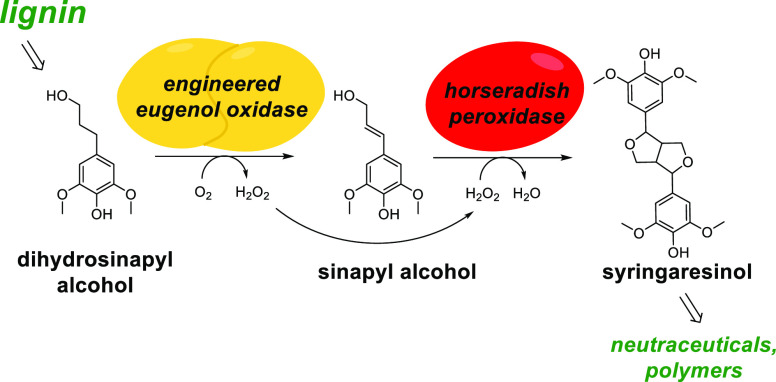

The drive for a circular bioeconomy has resulted in a great demand for renewable, biobased chemicals. We present a one-pot biocatalytic cascade reaction for the production of racemic syringaresinol, a lignan with applications as a nutraceutical and in polymer chemistry. The process consumes dihydrosinapyl alcohol, which can be produced renewably from the lignocellulosic material. To achieve this, a variant of eugenol oxidase was engineered for the oxidation of dihydrosinapyl alcohol into sinapyl alcohol with good conversion and chemoselectivity. The crystal structure of the engineered oxidase revealed the molecular basis of the influence of the mutations on the chemoselectivity of the oxidation of dihydrosinapyl alcohol. By using horseradish peroxidase, the subsequent oxidative dimerization of sinapyl alcohol into syringaresinol was achieved. Conditions for the one-pot, two-enzyme synthesis were optimized, and a high yield of syringaresinol was achieved by cascading the oxidase and peroxidase steps in a stepwise fashion. This study demonstrates the efficient production of syringaresinol from a compound that can be renewed by reductive catalytic fractionation of lignocellulose, providing a biocatalytic route for generating a valuable compound from lignin.

## Introduction

Recent studies have shown that lignin can serve as a viable starting material for new products. In recent years, a promising “lignin-first” strategy has been enabled by the emergence of so-called reductive catalytic fractionation. This methodology allows for the depolymerization of lignin prior to carbohydrate valorization.^1,2^ The process prevents lignin repolymerization by combining lignin conversion with biomass fractionation under reductive conditions, resulting in high yields of phenolic monomers from lignocellulose.^3−5^ This development calls for the development of new methods for the valorization of the phenols and other small molecules obtainable via the reductive catalytic fractionation of lignocellulose.^6−8^ In this context, syringaresinol, a symmetric lignan, is gaining considerable attention. This molecule has been extensively investigated for its medical properties as a bioactive compound.^9^ It has been suggested to lower oxidative stress by regulation of various signaling and antioxidant pathways.^10,11^ Moreover, as observed for other lignans, dietary syringaresinol modulates the composition of gut microbiota to promote health.^12,13^ Research on syringaresinol-derived glucosides revealed that they have multiple functions, including inhibition of tobacco mosaic virus replication^14^ and anxiolytic properties.^10^ Besides these potential health-related applications, syringaresinol has above all gained considerable interest as an excellent alternative to petroleum-based bisphenol A. The latter compound has been widely used as a comonomer in the production of various polymers.^15^ However, it is nowadays classified as an endocrine disruptor and is prohibited in many applications.^16^ Studies have revealed the potential of syringaresinol as a replacement for bisphenol A as a monomer, being used to generate polymers with useful thermal and thermomechanical properties.^17,18^

**Figure 1:**
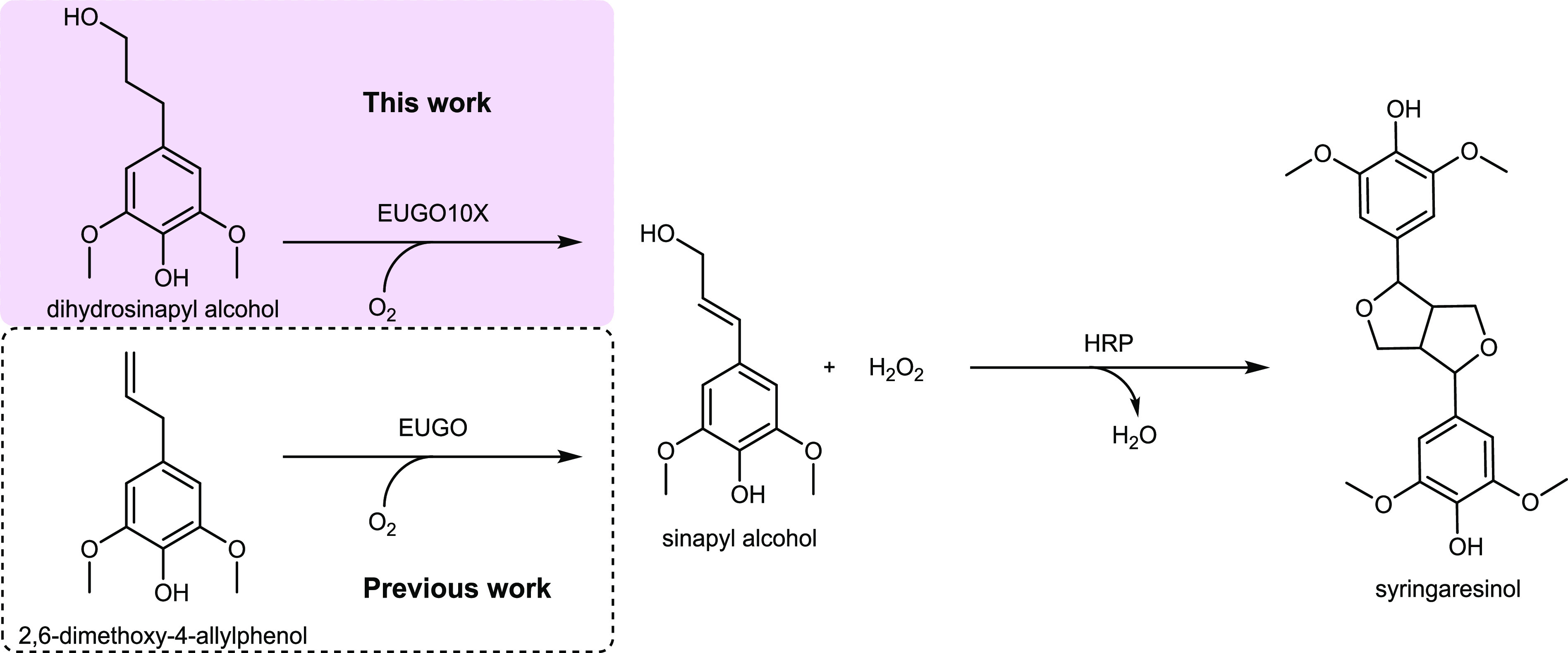
Scheme of the biocatalytic synthesis of syringaresinol from lignin-derived phenolic compounds.

A drawback of natural syringaresinol is its low yield of extraction from plants, and its chemical production has also been shown to be inefficient.^9,19^ The biocatalytic synthesis of syringaresinol has been therefore investigated, as this would allow for a selective, efficient, and eco-friendly process. Previously, we combined a flavoprotein oxidase, eugenol oxidase (EUGO), with horseradish peroxidase (HRP) to achieve a facile one-pot synthesis of lignin-like oligomers.^20^ Specifically, syringaresinol was synthesized via a biocatalytic double oxidation process, starting from 2,6-dimethoxy-4-allylphenol and proceeding via a sinapyl alcohol intermediate (Figure 1).^21^ While present in plant material, this starting material is not abundant, limiting the applicability of this route. Peroxidases and laccases^22^ have also been thoroughly investigated for the production of syringaresinol through the direct oxidative dimerization of sinapyl alcohol.^9,19^ However, this compound is too expensive to use in a cost-effective biocatalytic process. By contrast, dihydrosinapyl alcohol would be an attractive precursor, as it is one of the dominant alkylphenols obtainable through the reductive catalytic fractionation of lignocellulose (Figure 1).^23^ Dihydrosinapyl alcohol differs from sinapyl alcohol only by unsaturation of the alkyl substituent *para* to the phenol −OH. In previous work, we showed that a member of the vanillyl-alcohol oxidase (VAO) subfamily of flavoprotein oxidases can be engineered to catalyze the chemoselective dehydrogenation of 4-alkylphenols structurally similar to dihydrosinapyl alcohol (Figure S1).^24^

On these grounds, we identified the same enzyme, EUGO from *Rhodococcus jostii* RHA1, as a promising candidate biocatalyst for the efficient and selective dehydrogenation of dihydrosinapyl alcohol into sinapyl alcohol. This VAO-type enzyme is easy to produce using *Escherichia coli*, and its crystal structure has been determined. However, it also exhibits several key issues, including moderate stability, moderate conversion of the dihydrosinapyl alcohol substrate, and imperfect chemoselectivity (Figure S1). Aided by structural analysis and computational methods, we set out to engineer EUGO into a selective and robust catalyst for the dehydrogenation of dihydrosinapyl alcohol and use this biocatalyst in a cascade reaction to arrive at syringaresinol from dihydrosinapyl alcohol-containing materials.

## Materials and Methods

### Chemicals, Reagents, and Strains

Dihydrosinapyl alcohol, sinapyl alcohol, dihydroconiferyl alcohol, and coniferyl alcohol were purchased from Sigma-Aldrich and Fluorochem Limited. Syringaresinol analytical standard was previously synthesized by a EUGO-HRP cascade reaction and further purified by chromatography (SiO_2_, dichloromethane/methanol = 99:1) as previously described.^21^ Its purity was verified by ^1^H and ^13^C NMR.

Peroxidase from horseradish (HRP, product No. 77332, 150 U mg^–1^ based on activity with 2,2′-azino-bis(3-ethylbenzothiazoline-6-sulfonic acid at pH 6) was purchased from Sigma-Aldrich on February 10, 2022 and stored at 4 °C for further use. All components related to the medium and antibiotics were from Fisher Scientific chemicals. Solvents were purchased from Biosolve B.V. and Macron Fine Chemicals. Oligonucleotide primers were purchased from Sigma-Aldrich. PfuUltra II HotStart PCR (Polymerase) master mix from Agilent Technologies was used for generating the QuikChange mutations. QIAprep Spin Miniprep Kit from QIAGEN was applied to extract plasmids, and DNA sequencing was provided by Eurofins Genomics. SYPRO Orange protein stain (5000x concentrated in DMSO) was obtained from Thermo Fisher Scientific. The expression plasmid *pBAD-EUGO-His6x* was used, resulting in the expression of EUGO fused with a C-terminal His-tag through induction by l-arabinose. Unless stated otherwise, all of the single point mutants and combined mutants were prepared using *pBAD-EUGO-His6x* as the basis.*E. coli*NEB10-beta (New England Biolabs) was used as the host strain to express recombinant EUGO. EUGO and its variants with a C-terminal His-tag were purified as previously described and used for all experiments, except for the crystallization experiments. In that case, in order to be able to cleave the tag, EUGO8X and EUGO10X were expressed using the *pBAD-His6x-Sumo-EUGO* vector.

### Engineering of Eugenol Oxidase

Single mutants were created using the expression plasmid encoding wild-type EUGO as template and were screened as cell-free extracts. The screening of dihydroconiferyl alcohol/dihydrosinapyl alcohol conversions by cell-free extracts and purified enzymes was performed in reaction mixtures (500 μL) containing dihydroconiferyl alcohol or dihydrosinapyl alcohol (5 mM), EUGO-harboring cell-free extract (250 μL), potassium phosphate buffer (50 mM, pH 7.5), and DMSO (10%) at 25 °C with shaking at 150 rpm. Reactions were initiated by the addition of enzyme. Cell-free extracts harvested from 10 mL of bacterial culture were prepared in potassium phosphate buffer (50 mM, pH 7.5, and 1 mL). The same procedure was applied for screening reactions with purified enzymes, except that the concentration of biocatalyst was set to 10 μM. Aliquots (20 μL) were taken at various time intervals and quenched by addition of four volumes of acetonitrile prior to centrifugation to pellet precipitated protein. The supernatant was collected and analyzed on HPLC with an XSelect CSH fluoro-penyl column (130 Å, 5 μm, 4.6 mm × 250 mm, with a precolumn of the same material). The solvents used were (A) potassium phosphate buffer (12 mM, pH 7) and (B) HPLC-grade acetonitrile. The HPLC method was a gradient of 25–60% buffer B over 6 min, 60% buffer B for 0.5 min, followed by a 1.5 min gradient of 60–25% and re-equilibration for 1 min. The absorbance of the eluent was recorded at 280 nm (Figures S8 and S9).

### Expression and Purification of EUGO8X and EUGO10X Mutants

The expression of the EUGO mutants was performed by growing single colonies (*pBAD-His-Sumo-EUGO8X* and *EUGO10X*) in LB medium supplemented with ampicillin (100 μg mL^–1^) at 37 °C overnight. These precultures were then used to inoculate Terrific Broth cultures (1 L), which were then incubated at 37 °C with shaking at 200 rpm, until OD_500_ reached 0.7–0.8. Enzyme expression was then induced by the addition of l-arabinose (0.02% w/v), and cultures were shaken for a further 20 h at 24 °C.

For enzyme purification, cells were harvested by centrifugation (6,000*g*, 15 min, 10 °C) and the pellet was resuspended in lysis buffer (50 mM Tris-HCl pH 8.0, 150 mM NaCl, 5 mM imidazole, 1 mg mL^–1^ lysozyme, 10 μM FAD), including phenylmethylsulfonyl fluoride (1 mM), leupeptin (10 μM), pepstatin (10 μM), and DNase I (0.02 mg mL^–1^). Cells were lysed by sonication. Lysed cells were centrifuged (56,000*g*, 1 h, 4 °C), and the supernatant was collected and filtered (0.45 μm) prior to being loaded onto the HisTrap HP column (5 mL of resin, Cytiva), pre-equilibrated with Buffer A (50 mM Tris-HCl pH 8.0, 150 mM NaCl, 5 mM imidazole). The His-tagged protein was eluted with elution buffer (50 mM Tris-HCl (pH, 8.0), 150 mM NaCl, 300 mM imidazole) and concentrated to a final volume of 1 mL. For crystallization studies, the SUMO-tag was removed. For this, the protein was incubated with 6xHis-tagged SUMO protease (1.0 mg mL^–1^) with overnight dialysis using a 10 kDa dialysis cassette (Thermofisher) to remove imidazole. After buffer exchange, the protein was then loaded onto a HisTrap column (5 mL, Cytiva) to perform a reverse-nickel purification. The column was pre-equilibrated with Buffer A, with the protein eluting immediately. The tagless protein was concentrated to a final volume of 500 μL and incubated with FAD (1 mM) overnight at 4 °C. The day after, the sample was loaded onto a gel filtration column (Superdex 200 10/300, Cytiva) pre-equilibrated with Tris-HCl buffer (10 mM, pH 7.5 at 4 °C). The sample was then eluted with the same buffer, affording EUGO with very high purity and homogeneity.

### Crystallization of EUGO8X and EUGO10X Mutants

Purified EUGO8X was concentrated by centrifugal filtration to 15 mg mL^–1^ in a Tris-HCl buffer (10 mM, pH 7.5, 4 °C). Crystallization was performed using the microbatch technique at 20 °C by mixing equal volumes of protein and precipitant solution consisting of MgCl_2_/CaCl_2_ (0.06 M), sodium HEPES/MOPS (0.1 M), ethylene glycol (20%), and PEG 8000 (20% w/v). After 3 days, rhombic yellow crystals were obtained. They were soaked for 45 min in a cryoprotection solution corresponding to the crystallization conditions with the addition of dihydrosinapyl alcohol (5 mM). Crystals of EUGO10X mutant were grown at 20 °C by the sitting-drop vapor diffusion method. Protein (17 mg mL^–1^) was mixed with an equal volume of a precipitant solution consisting of Tris-HCl (0.1 M, pH 8.0, 28% PEG 8000). After one month, yellow rod-shaped crystals were obtained and soaked for 1 h in a reservoir solution with dihydrosinapyl alcohol (5 mM). X-ray diffraction data used for structure determination and refinement were collected at the PXIII beamline of the Swiss Light Source in Villigen (SLS, Switzerland) and at the Massif1 beamline of the European Synchrotron Radiation Facility (ESRF, Grenoble). The crystal structures were solved by molecular replacement using the coordinates of EUGO from *Rhodococcus jostii* RHA1 (PDB entry 5FXD) as a search model excluding the ligand and water molecules. Crystallographic computing, manual building, the addition of waters, and crystallographic refinement were performed with COOT^25^ and Phenix.^26^ Figures were created with PyMOL (DeLano Scientific; www.pymol.org) and Chimera.^27^ Crystallographic statistics are listed in Table S2.

### Biocatalytic One-Pot Synthesis of Syringaresinol from Dihydrosinapyl Alcohol

Relevant compounds were analyzed by HPLC (Figure S2) as described above, and calibration curves were established in the range of 0.01 to 10 mM (Figure S3). The alcohol hydroxylation products (1-(4-hydroxy-3-methoxyphenyl)propane-1,3-diol and 1-(4-hydroxy-3,5-dimethoxyphenyl)propane-1,3-diol) and ketone products (3-hydroxy-1-(4-hydroxy-3-methoxyphenyl)propan-1-one and 3-hydroxy-1-(4-hydroxy-3,5-dimethoxyphenyl)propan-1-one) of the oxidations of coniferyl and sinapyl alcohols were not commercially available.

The one-pot reactions were performed with dihydrosinapyl alcohol (5 mM), EUGO10X (10 μM), HRP (0.01–20 μM) in potassium phosphate buffer (50 mM, pH 7.5, 500 μL), and DMSO (10%) at a variety of temperatures, with shaking at 150 rpm in a 4 mL glass vial. To complete the synthesis of syringaresinol in a stepwise manner, the reactions were incubated at 35 °C without HRP for 3 h prior to addition of HRP of various concentrations from a 200 μM stock solution.

For monitoring of the formation of intermediate sinapyl alcohol and product syringaresinol, samples (20 μL) were quenched with four volumes of acetonitrile and centrifuged at 14,000 rpm for 5 min. The supernatant was analyzed using a JASCO HPLC system. Sample (10 μL) was injected onto a XSelect CSH Fluoro-Phenyl Column (130 Å, 5 μm, 4.6 mm × 250 mm, with a precolumn of the same material) and analyzed by the same method as described above.

For reaction scale-up, dihydrosinapyl alcohol (5 mM from a 500 mM stock in acetonitrile, 42 mg) was incubated with EUGO10X (10 μM) in potassium phosphate buffer (50 mM, pH 7.5, 40 mL) in a glass conical flask at 45 °C for 3 h with shaking at 100 rpm. The reaction was initiated by the addition of substrate. At this point, lyophilized HRP (10 mg) was added, and the mixture was shaken for a further 21 h under the same conditions. The reaction was divided in half and quenched by addition of four volumes of acetonitrile, and the solvents were removed as an azeotropic mixture by rotary evaporation. The solid residue was suspended in ethyl acetate, then filtered, and evaporated, leaving an oily brown residue (7.2 mg, 36% yield). The oily product was analyzed by NMR and mass spectrometry and found to be almost pure syringaresinol. The intermediate mixture of starting material and sinapyl alcohol was isolated via a 3 h reaction at 25 °C with the same constituents (20 mL), prior to a similar workup and analysis.

## Results and Discussion

### Engineering of Eugenol Oxidase

As a first step to identify mutations that improve the selectivity of EUGO toward dehydrogenation of the target compound, a small library of 26 single enzyme mutants was prepared (Table S1). Similarly to a previous study,^24^ the mutations were selected based on a structural analysis of EUGO that included the use of Rosetta to probe their effect on substrate binding. Most of the targeted residues are in the substrate-binding pocket. Enzyme variants were tested as cell-free extracts, and conversions were assayed after 72 h of incubation with dihydrosinapyl alcohol (5 mM).

**Figure 2:**
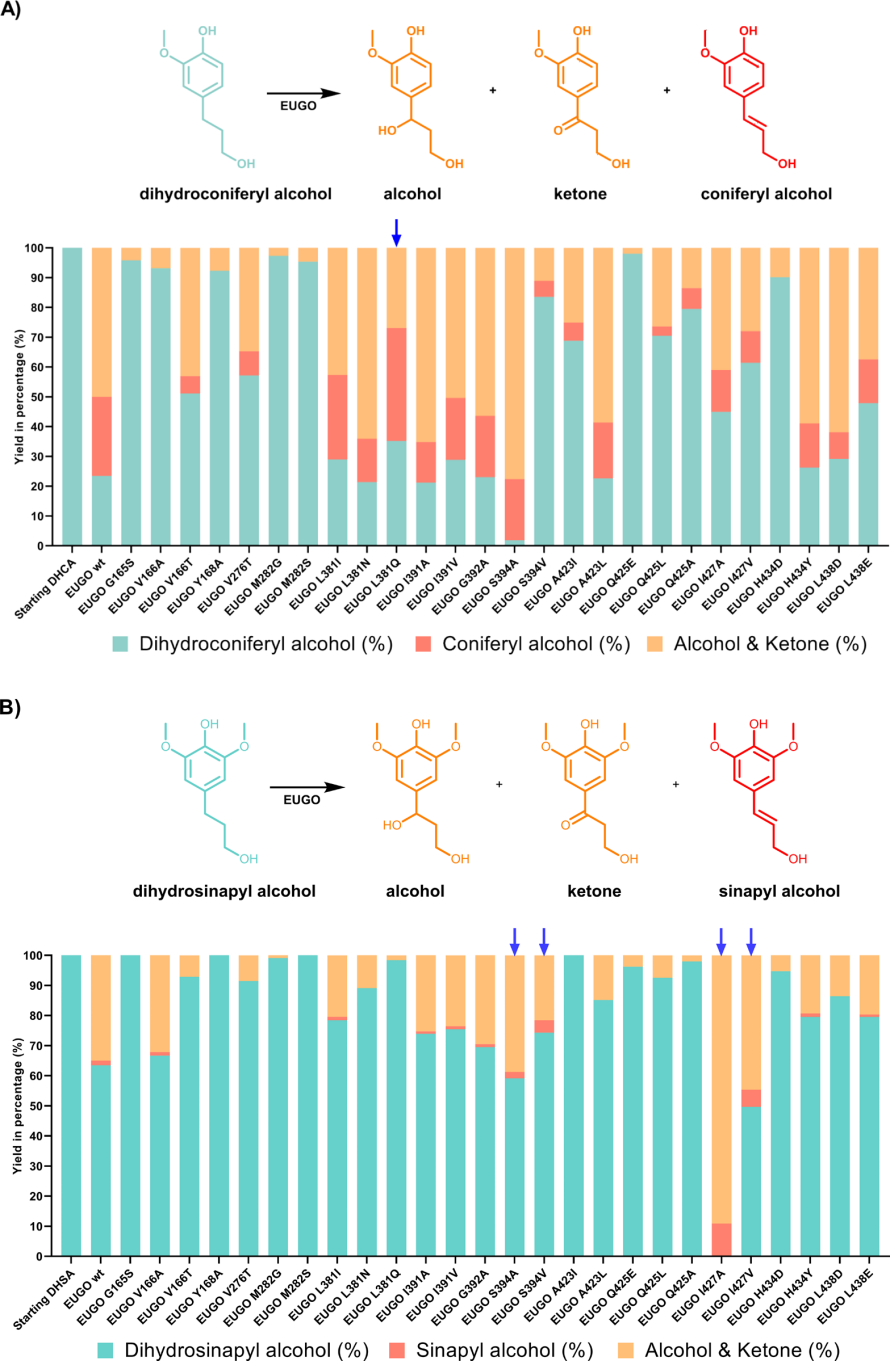
Mutant screening for enzymatic conversion of dihydroconiferyl alcohol and dihydrosinapyl alcohol. Dihydroconiferyl alcohol/dihydrosinapyl alcohol, dehydrogenation products coniferyl alcohol/sinapyl alcohol, and the corresponding oxygenation products (alcohol/ketones) are shown together in cyan, red, and orange, respectively. Mutants of interest are marked with blue arrows. (A) 24 h conversion of dihydroconiferyl alcohol by EUGO single mutants using cell-free extracts; (B) 72 h conversion of dihydrosinapyl alcohol by EUGO single mutants using cell-free extracts. Conversions of dihydroconiferyl alcohol or dihydrosinapyl alcohol (5 mM) were carried out in KPi buffer (50 mM, pH 7.5).

The two *ortho*-methoxy groups on the aromatic ring of dihydrosinapyl alcohol make it a sterically challenging substrate for wild-type EUGO.^21^ For reference, the mutants were also screened against dihydroconiferyl alcohol, which is identical to dihydrosinapyl alcohol, except that its aromatic ring is substituted with only one methoxy group at the *ortho* positions. Dihydroconiferyl alcohol is a less challenging substrate for EUGO, and it was hypothesized that it may be easier to find useful mutants or hotspots when screening against this simpler compound. The molecular volume of dihydroconiferyl alcohol (178 Å^3^)^28^ is substantially less than that of dihydrosinapyl alcohol (206 Å^3^), even though the two compounds feature the same 1-propanol side chain (Figure 2A,B).

HPLC analyses revealed that wild-type EUGO efficiently converts dihydroconiferyl alcohol in 24 h (77% conversion), with with 27% of the starting material being converted to the desired dehydrogenation product, coniferyl alcohol (35% chemoselectivity). Against dihydrosinapyl alcohol, however, less than 40% conversion was achieved after a substantially longer 72 h incubation, generating only <2% sinapyl alcohol (4% chemoselectivity; Figure 2A,B). The remainder comprised the respective alcohol and ketone “hydroxylation pathway” byproducts (Figure S1).

The screen of the initial library revealed a few mutants with improved properties (Figure 2A,B). Specifically, the L381Q mutation enhanced the selectivity of EUGO for coniferyl alcohol dehydrogenation, exhibiting 58% chemoselectivity, an improvement of 18% over wild-type, albeit with slightly lower conversion. Despite the improved performance against the smaller substrate, EUGO L381Q was ineffective against dihydrosinapyl alcohol, exhibiting <2% conversion and no detectable formation of sinapyl alcohol. As well as this, the mutation S394A was identified, which afforded superior conversion of coniferyl alcohol (>98%) but marginally reduced chemoselectivity.

Conversely, two mutations at I427 (I427A and I427V) substantially improved the rate of conversion of dihydrosinapyl alcohol (>99% for I427A after 72 h) and the selectivity for dehydrogenation (11%) but reduced activity against dihydroconiferyl alcohol. EUGO S394A promoted a slightly improved conversion of dihydrosinapyl alcohol and marginally improved chemoselectivity, while the mutant S394V achieved 16% chemoselectivity (12% better than wild-type) but with slightly reduced conversion. Notably, both S384A and S394V were identified in a previous study improving the chemoselectivity of the dehydrogenation of 4-propylguaiacol.^24^ From these results, L381, S394, and I427 were identified as mutagenesis hotspots.

**Figure 3:**
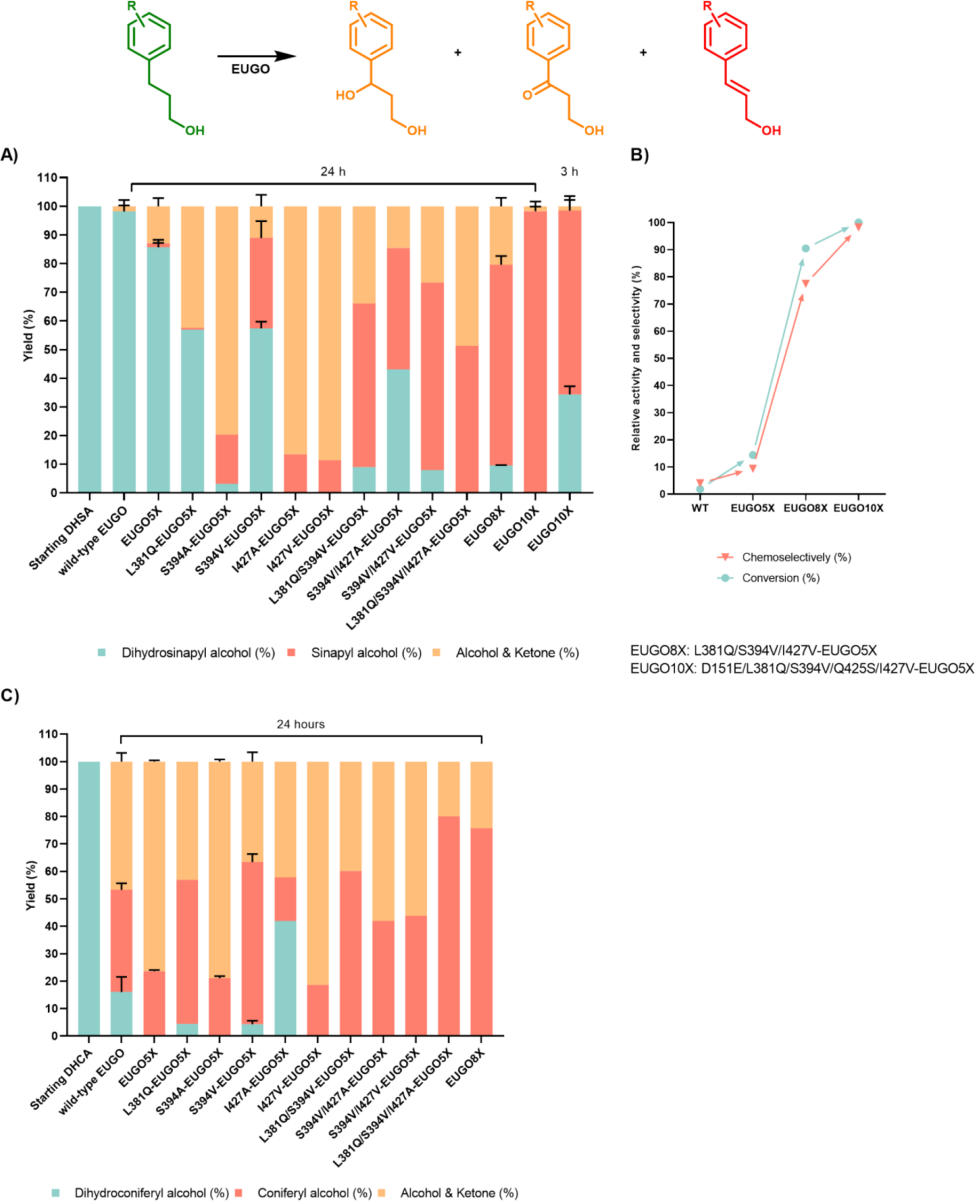
Screening of EUGO 5X single, double, and triple mutants for the conversion of dihydrosinapyl alcohol and dihydroconiferyl alcohol. Dihydrosinapyl alcohol/dihydroconiferyl alcohol, sinapyl alcohol/coniferyl alcohol, and their corresponding alcohols and ketones together are shown in cyan, red, and orange, respectively. (A) Conversions of dihydrosinapyl alcohol (5 mM) were carried out in the presence of purified enzymes (10 μM) in a KPi buffer (50 mM, pH 7) with DMSO (10%) and analyzed by HPLC. (B) Evolution of eugenol oxidase for chemoselective oxidation of dihydrosinapyl alcohol. Using the 24 h conversion of dihydrosinapyl alcohol by EUGO10X as reference, the relative activity and selectivity for substrate oxidation were compared among wild-type and a few mutants. (C) Conversions of dihydroconiferyl alcohol (5 mM) were carried out in the presence of purified enzymes (10 μM) in KPi buffer (50 mM, pH 7.5) with DMSO (10%) and analyzed by HPLC.

The mutations identified from the initial screen, L381Q, S394A, S394V, I427A, and I427V were next individually incorporated into a thermostable variant of EUGO (EUGO S81H A423M H434Y I445D S518P, referred to hereafter as EUGO5X; *T*_m_ of 80 °C) that was obtained previously using the FRESCO algorithm.^24^ The reactivity of EUGO5X against the two substrates was similar to that of the wild type but with marginally improved conversion against both substrates (Figure 3A).

Rewardingly, EUGO5X S394A, I427A, and I427V were found to be especially effective, converting all or nearly all of the target substrate dihydrosinapyl alcohol in only 24 h. EUGO5X L381Q also improved the conversion of dihydrosinapyl alcohol but reduced selectivity for dehydrogenation, in contrast with the reaction with dihydroconiferyl alcohol (Figure 3A). EUGO5X S394V displayed significantly improved chemoselectivity for dehydrogenation (70%), but at the expense of less conversion than other mutants (Figure 3A). As selectivity was the priority for engineering, rather than conversion/yield, using the expression plasmid encoding EUGO5X S394V was used as a template for the next round.

Several EUGO5X S394V double mutants were next prepared, which incorporated the conversion-boosting mutations L381Q, I427A, and I427V. The mutants were tested as purified proteins in reactions lasting 24 h, instead of the previous incubations of 72 h. EUGO5X L381Q S394V and EUGO5X S394V I427V both converted 90% of dihydrosinapyl alcohol, an improvement on EUGO5X S394V, with 62 and 71% chemoselectivity for dehydrogenation, respectively. EUGO5X S394V I427A exhibited a reduced conversion (57%) but slightly improved chemoselectivity (74%).

As all of the tested double mutants of EUGO5X proved to be beneficial, the two possible triple mutants were next prepared. EUGO5X L381Q S394V I427V (“EUGO8X” hereafter) was more selective than EUGO5X L381Q S394V I427A, converting 90% of dihydrosinapyl alcohol with 77% chemoselectivity over 24 h (Figure 3A-B). Additionally, the combination of selected mutations presented a similar enhancement of the selective conversion of dihydroconiferyl alcohol into coniferyl alcohol (Figure 3C). Despite this, however, the EUGO5X triple mutant was still not a perfect dihydrosinapyl alcohol dehydrogenase.

For the final push toward complete conversion and selectivity, we harkened back to our previous engineering campaign against EUGO, in which we identified such a biocatalyst for the dehydrogenation of the related substrate 4-propylguaiacol.^24^ In that campaign, mutations D151E and Q425S were found to be critical for controlling the selectivity and rate of dehydrogenation, respectively. We therefore incorporated these two mutations into EUGO8X, generating a new biocatalyst referred to as EUGO10X (Figure 3A,B). When purified and assayed, the EUGO10X biocatalyst generated sinapyl alcohol from dihydrosinapyl alcohol with only a trace amount of side products and with complete conversion over 24 h. This signified the success of our engineering campaign. The inclusion of L381Q in the sequence of the final biocatalyst vindicated our strategy of screening mutants against the simpler model substrate, dihydroconiferyl alcohol; if not for this secondary screen, this mutant would have been discarded in the beginning due to the low activity of EUGO L381Q against dihydrosinapyl alcohol.

To probe its thermostability, EUGO10X was also analyzed using the ThermoFluor assay. The 10-fold mutant was found to have a melting temperature of 76 °C, only slightly below that of the thermostable EUGO5X template (80 °C).

### Crystal Structures of Engineered Oxidases

**Figure 4:**
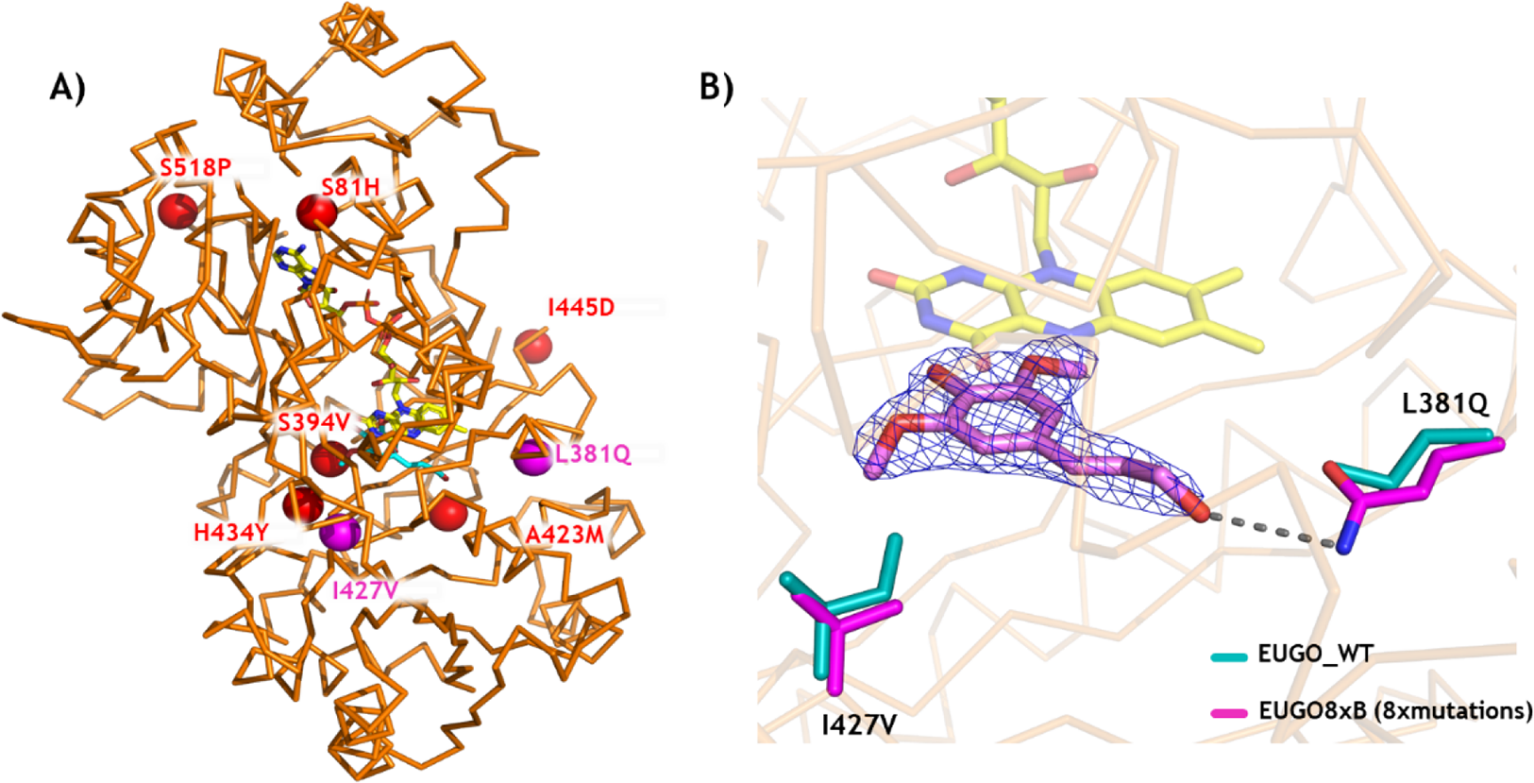
X-ray crystal structure of the 8-fold mutant, EUGO8X. (A) Backbone of EUGO8X with its mutation sites (spheres). The active-site mutations I427V and L381Q are in magenta. The FAD is shown in yellow sticks. (B) Comparison of active sites of EUGO8X (magenta) and wild-type EUGO (cyan; PDB code 5FXD). The 2Fo-Fc electron density map of sinapyl alcohol is contoured at a 1.2 σ level.

To understand the effects of the introduced mutations in the EUGO8X and EUGO10X, their crystal structures were solved at 2.3 and 1.6 Å resolution, respectively. By soaking the crystals with substrate, we obtained the structures in complex with dihydrosinapyl alcohol. Both catalysts contain five stabilizing mutations (S81H, A423M, H434Y, I445D, and S518P), as well as S394V, which have already been discussed in our previous engineering study of EUGO for the dehydrogenation of 4-propylguaiacol (Figure 4A).^24^ Focusing on the hereby identified activity-improving mutations, the crystal structures show that I427V introduces more space for accommodating the second *ortho*-methoxy group of dihydrosinapyl alcohol relative to wild-type, explaining the improved conversions allowed by this mutation (Figure 4B). L381Q meanwhile forms a new hydrogen bond with the hydroxy group of the substrate side chain, thereby forming a specific substrate–protein interaction (Figure 4B). The combined effect of these mutations rationalizes the enhanced activity of the mutant toward dihydrosinapyl alcohol.

**Figure 5:**
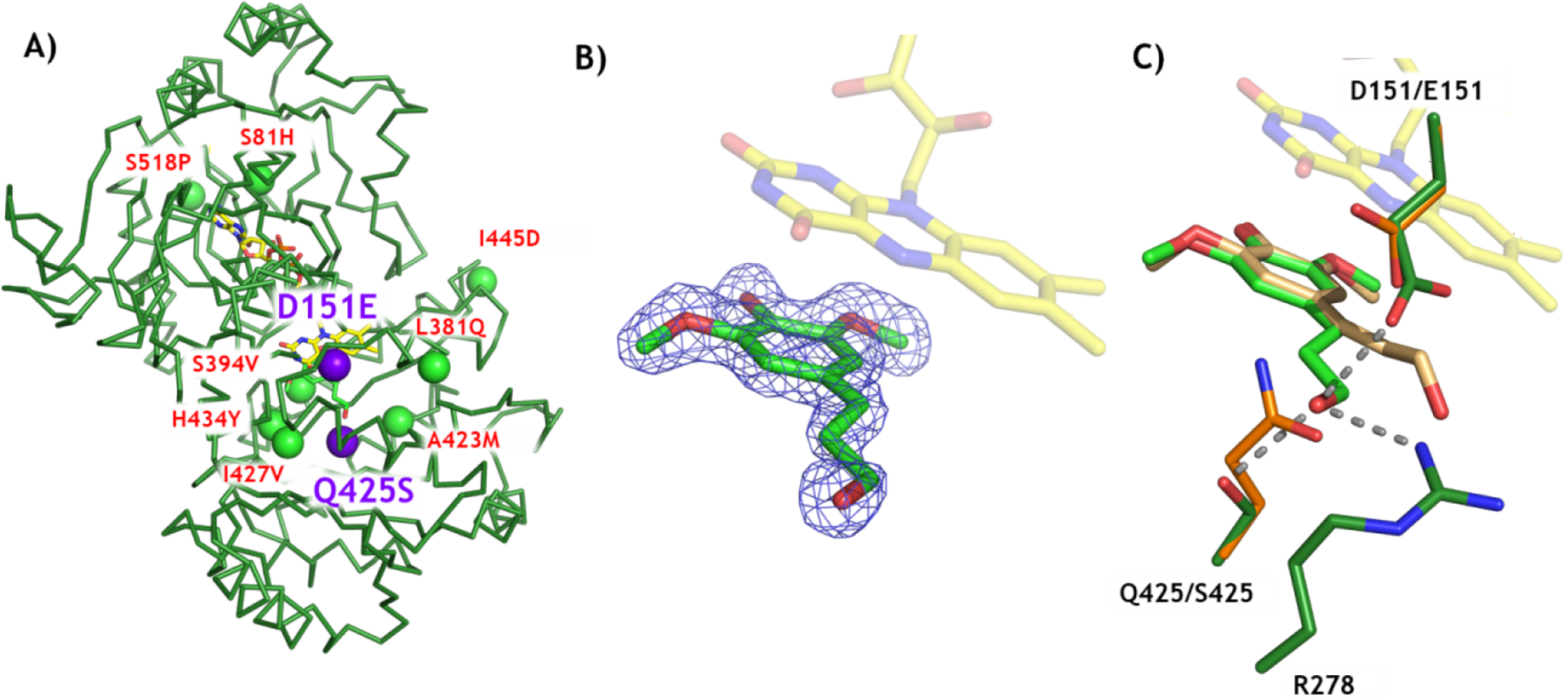
X-ray structure of EUGO10X. (A) Backbone of EUGO10X with its mutation sites shown as spheres. The two additional chemoselectivity-affording mutations are represented with purple spheres (D151E and Q425S). (B) 2Fo-Fc electron density map of dihydrosinapyl alcohol in the active site. The map is contoured at a 1.0 σ level. (C) Comparison between EUGO8X (orange) and EUGO10X (green). The bound ligands are shown in wheat (EUGO8X) and green (EUGO10X).

As explained above, the D151E and Q425S mutations were introduced in the heart of the active site of EUGO8X, resulting in the EUGO10X mutant featuring enhanced chemoselectivity for dehydrogenation (Figure 5A). The high-quality electron density map of EUGO10X complexed with dihydrosinapyl alcohol clearly defines the binding pose of the substrate in the active site (Figure 5B). By superposing the substrate-bound structures of EUGO8X and EUGO10X, a subtle ∼20° rotation of the plane of the aromatic ring and concomitant shift in the position of the propanol side chain of dihydrosinapyl alcohol can be observed (Figure 5C). This is permitted by the smaller side chain of Q425S. Improved substrate binding is also allowed by the mutations, via a hydrogen-bonding network of the substrate and the side chains of D151E and R278 (Figure 5C). Inspection of the crystal structure further reveals that E151 may be better positioned than D151 to promote the deprotonation of the reactive quinone methide intermediate (Figure S1), leading to the formation of the dehydrogenated product. Furthermore, E151 may selectively decrease the accessibility of the side chain of the *para*-quinone methide intermediate to water, due to the bulkiness of its side chain,^24^ causing the drastic reduction of the rate of formation of hydroxylated side-products (Figure 3A).

### One-Pot Two-Enzyme Conversion of Dihydrosinapyl Alcohol into Racemic Syringaresinol

**Figure 6:**
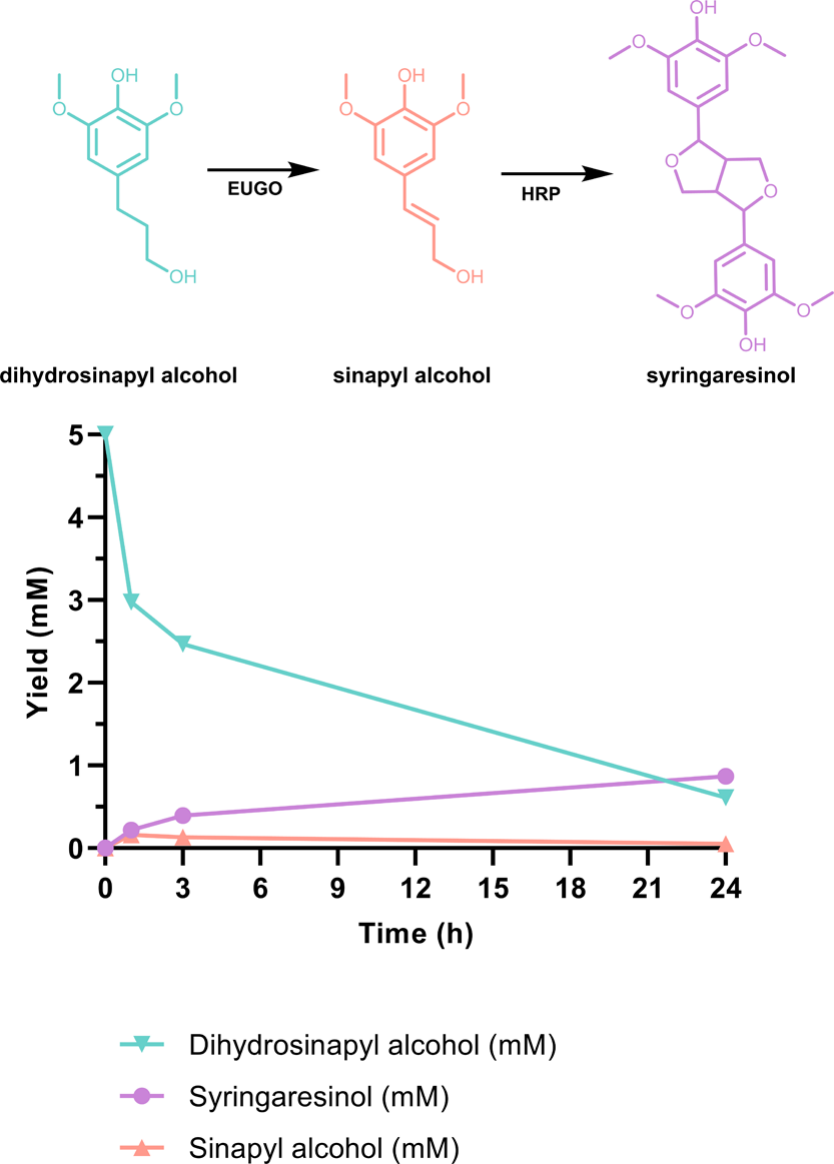
Time-course monitoring of one-pot conversion of dihydrosinapyl alcohol to syringaresinol by an oxidase-HRP cascade reaction. Dihydrosinapyl alcohol, sinapyl alcohol, and syringaresinol are displayed in the cyan, orange, and violet lines, respectively. The conversion of dihydroconiferyl alcohol (5 mM) was carried out in the presence of EUGO10X (10 μM) and HRP (10 μM) in KPi (50 mM, pH 7.5) with DMSO (10%), 25 °C.

Having engineered a biocatalyst for the selective dehydrogenation of dihydrosinapyl alcohol, we set out to combine it with a peroxidase for a one-pot biocatalytic synthesis of syringaresinol. HRP was employed for the oxidative coupling of oxidase-generated sinapyl alcohol into syringaresinol. In this cascade, H_2_O_2_ liberated as a byproduct of the EUGO10X-catalyzed dehydrogenation reaction serves as a substrate for HRP (Figure 1). The one-pot synthesis of syringaresinol was initially attempted with EUGO10X and HRP (10 μM each) and dihydrosinapyl alcohol (5 mM) in potassium phosphate buffer (50 mM, pH 7.5) with DMSO (10%) at 25 °C. The reaction was monitored over time by HPLC. After 24 h, most of the dihydrosinapyl alcohol had been converted, and a significant amount of syringaresinol was indeed formed (Figure 6). However, the analysis revealed some byproducts, limiting the yield of syringaresinol to 40%. These byproducts may be the result of unselective oligomerization of sinapyl alcohol and other phenolic compounds by HRP^20,29^ and/or their subsequent depolymerization.^30−32^

**Figure 7:**
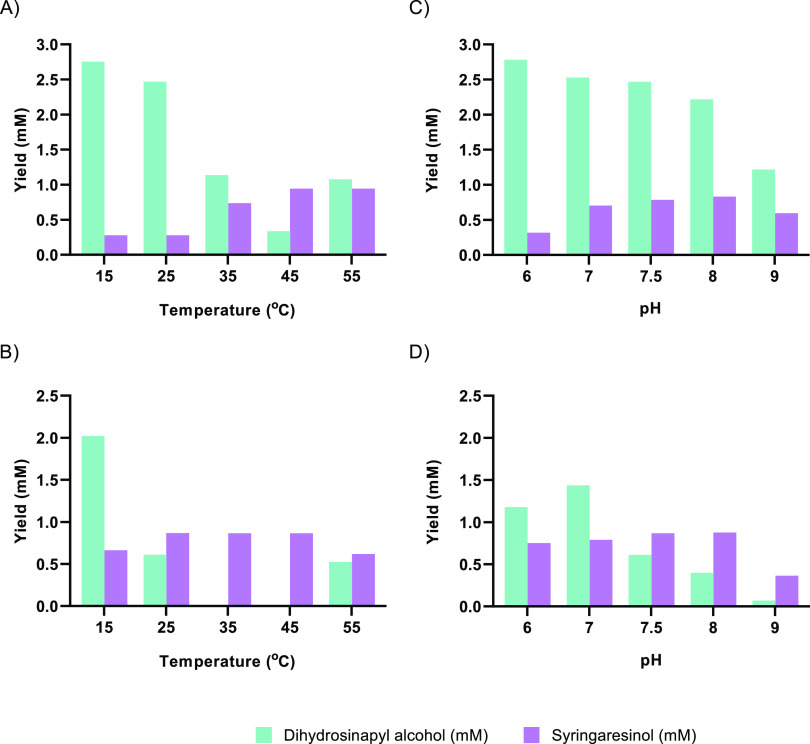
Effects of temperature and pH on conversion of dihydrosinapyl alcohol to syringaresinol by a EUGO10X-HRP cascade reaction. The substrate, dihydrosinapyl alcohol, and final product, syringaresinol, are displayed in cyan and violet, respectively. Conversions of dihydrosinapyl alcohol (5 mM) were carried out using EUGO10X (10 μM) and HRP (10 μM) in KPi buffer (50 mM, pH 7.5) with DMSO (10% v/v) at the indicated temperatures over (A) 3 and (B) 24 h. Conversions of dihydroconiferyl alcohol (5 mM) were carried out using EUGO10X (10 μM) and HRP (10 μM) in KPi buffer (50 mM, pH 6, 7 and 7.5) or Tris (pH 8 and 9) with DMSO (10%) at 25 °C over (C) 3 and (D) 24 h. The theoretical maximum yield of syringaresinol is 2.5 mM.

**Figure 8:**
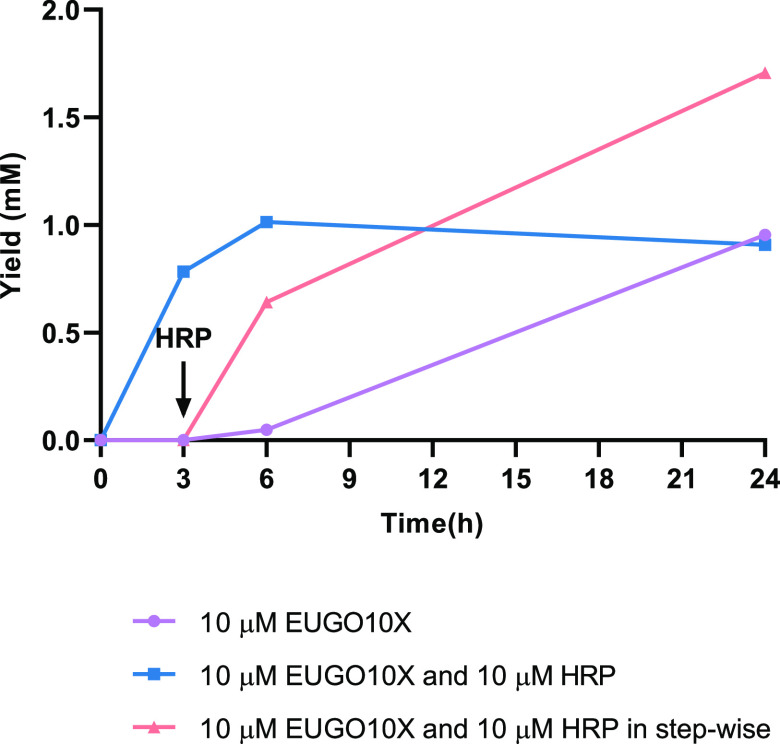
Effect of amount of HRP in the formation of syringaresinol in a EUGO10X-HRP cascade reaction starting from dihydrosinapyl alcohol. The one-pot conversions with no HRP (purple) and 10 μM HRP (blue), as well as a delayed addition of 10 μM HRP (orange) are shown.

The effects on the reaction of the temperature and pH were next studied. Temperature strongly influenced the conversion of dihydrosinapyl alcohol to syringaresinol. In the 15–45 °C range, higher temperatures improved the rate of syringaresinol formation but gave similar final yields after 24 h (Figure 7A,B). It appeared that reaction temperatures over 25 °C improved the rate of consumption of dihydrosinapyl alcohol but without a concomitant improvement in syringaresinol titer. At 55 °C, dihydrosinapyl alcohol consumption was inhibited. This temperature effect is in line with the reported optimum temperature of 50 °C for oxidative polymerization of monolignols by HRP.^33^ Additionally, dihydrosinapyl alcohol appeared to be slightly unstable in buffered aqueous media over 35 °C (Figure S4). Therefore, we decided to continue to use a temperature of 35 °C at an optimal value of pH 7.5 (Figure 7C,D). Using these conditions, a yield of about 40% syringaresinol was achieved in 3 h (Figure 8). Longer incubations only led to a reduced yield of syringaresinol, possibly due to HRP-catalyzed side-reactions. Supplementing the reaction mixture with H_2_O_2_ (1–10 mM) also did not help, actually reducing the yield of syringaresinol to 10% (Figure S5A). The reaction was also attempted in the absence of HRP. Interestingly, the final yield of syringaresinol after 24 h was almost identical with that of the reaction *with* HRP (40%, Figure 8), although the rate of syringaresinol formation was significantly reduced.

It appeared possible that dihydrosinapyl alcohol is an inhibitor or a substrate of HRP. We therefore attempted a stepwise reaction mode, in which HRP is added only after time has been allowed for the consumption of the majority of dihydrosinapyl alcohol starting material. When HRP was added to the reaction at the 3 h mark instead of at the beginning, significantly improved yields of syringaresinol were obtained (Figure 8). The concentration of HRP added (0.01–20 μM) had little effect on the outcome of the reaction after 24 h (Figure S5B). Addition of a combination of HRP and hydrogen peroxide also did not lead to a higher yield of syringaresinol. Nevertheless, using the optimized conditions and stepwise addition of biocatalysts, an analytical yield of 68% syringaresinol could be obtained (Figure 8). Cascade and dehydrogenation reactions were later performed at milligram scale, and the pure isolated syringaresinol product (7.2 mg, 37%) and dihydrosinapyl alcohol/sinapyl alcohol product mixture were characterized by NMR and mass spectrometry (Figures S6 and S7). The data obtained matched very closely with literature values.^21^ Therefore, EUGO10X has been demonstrated to be a promising biocatalyst for implementation in the cascade synthesis of the affordable and renewable substrate dihydrosinapyl alcohol into syringaresinol.

## Conclusions

Recent progress in chemical processes that can depolymerize lignin into alkylphenols, such as reductive catalytic fractionation, calls for the development of new processes that allow for the conversion of these phenols to valuable compounds. Syringaresinol is a valuable bioactive compound and a promising biobased building block for use in polymers. In this work, an efficient and selective oxidase biocatalyst was engineered, through exploiting a combination of computational and structural predictions, to catalyze the selective oxidation of dihydrosinapyl alcohol into sinapyl alcohol. Elucidation of the crystal structures of engineered oxidase variants revealed that subtle changes in the substrate binding pocket enable a switch in the substrate specificity. Furthermore, cascading the tailored oxidase with HRP enabled an effective biocatalytic synthesis of racemic syringaresinol. Conveniently, the oxidase generates hydrogen peroxide required by the peroxidase. Optimization of this cascade conversion revealed unexpected challenges in controlling the HRP-catalyzed oligomerization of phenolic compounds. The optimal setup for generating syringaresinol was found to be the sequential addition of the two biocatalysts, allowing EUGO10X to consume the bulk of the starting material before the addition of HRP. In this sequential setup, most of the formed sinapyl alcohol is converted into the lignin syringaresinol (68% yield). This approach allows facile one-pot conversion of lignin-derived dihydrosinapyl alcohol into a valuable lignan.

## Supporting Information Available

Mutations computationally predicted and rationally determined (Table S1), data collection and refinement statistics for EUGO8X and EUGO10X, two developed mutants (Table S2), HPLC analysis of chemicals relevant to biotransformation and their calibration curves (Figures S2 and S3), temperature and pH condition study (Figure 7), thermostability of dihydrosinapyl alcohol (Figure S4), condition optimization of biocatalytic synthesis of syringaresinol in one pot (Figure S5), NMR and mass spectrometry analysis of products (Figures S6 and S7), HPLC analysis of conversions of two substrates by EUGO variants (Figures S8 and S9), other details of materials used in this research, experimental procedures, and crystallization approach (PDF)

## ABBREVIATIONS

EUGO: eugenol oxidase
HRP: horseradish peroxidase
VAO: vanillyl-alcohol oxidase.
