# Supplementary material for: One-Pot Biocatalytic Synthesis of rac-Syringaresinol from a Lignin-Derived Phenol
Source: ACS Catal. 2023 Oct 31;13(22):14639–49. doi: 10.1021/acscatal.3c04399 (PMC10660655; doi:10.1021/acscatal.3c04399)
Supplement: Supplementary file 1 — cs3c04399_si_001.pdf [file cs3c04399_si_001.pdf]

Supporting Information

One-pot biocatalytic synthesis of *rac*-syringaresinol from a lignin-derived phenol

Yiming Guo†, Laura Alvigini‡, Mohammad Saifuddin†, Ben Ashley, Milos Trajkovic†, Lur Alonso-Cotchico§, Andrea Mattevi*,‡, and Marco W. Fraaije*,†

† Molecular Enzymology Group, University of Groningen, Nijenborgh 4, 9747 AG Groningen, the Netherlands

‡ Department of Biology and Biotechnology "Lazzaro Spallanzani", University of Pavia, Via Ferrata 9, 27100 Pavia, Italy

§ Zymvol Biomodeling S.L., Carrer Roc Boronat, 117, 08010, Barcelona, Spain

*Corresponding Authors

E-mail for M.W.F.: m.w.fraaije@rug.nl

E-mail for A.M.: andrea.mattevi@unipv.it

Abbreviations: BPA, bisphenol A; EUGO, eugenol oxidase; HRP, horseradish peroxidase, DHSA, dihydrosinapyl alcohol, DHCA, dihydroconiferyl alcohol.

Table S1. Predicted active site mutations.

Rosetta	V166A, V166T, Y168A, V276T, M282G, M282S, L381I, L381Q, I391A, I391V, G392A, S394A, S394V, A423L, Q425L, I427V
Rational	G165S, L381N, A423I, Q425E, I427A, H434D, H434L, L438D, L438E, I427G

Table S2. Data collection and refinement statistics for EUGO8X and EUGO10X.

	EUGO8X	***EUGO10X***
Space group	P1	$P\ 2_1\ 2_1\ 2_1$
Unit cell axes (Å)	113.56,142.99, 154.60	57.65, 98.05, 177.34
Unit cell angles (°)	114.87, 97.00, 93.28	90, 90, 90
Resolution (Å)	2.3	1.65
PDB code	8BAP	8BAM
$R_{sym}{}^{a,b}$	0.1592 (0.8421)	0.09249 (1.028)
$CC_{1/2}$	0.988 (0.547)	0.998 (0.674)
Completeness[b] (%)	98.21 (97.67)	99.41 (95.48)
Unique reflections	379400	120212
Redundancy	3.5 (3.6)	6.4 (6.3)
I/σ^{b}	7.2 (1.6)	13.8 (1.7)
N° of non-hydrogen atoms		
protein/FAD	68547/16x53	9917/2x53
ligand	16x15	2x15
water	1287	703
Average B value ($Å^2$)	34.51	23.43
$R_{crys}{}^{c}$ (%)	19.3	17.2
$R_{free}{}^{c}$ (%)	24.9	20.6
Rms bond length (Å)	0.009	0.007
Rms bond angles (°)	1.02	0.94

[a] $R_{sym}=\sum|I_i-\langle I\rangle|/\sum I_i$, where I_i is the intensity of ith observation and $\langle I\rangle$ is the mean intensity of the reflection.

[b] Values in parentheses are for reflections in the highest resolution shell.

[c] $R_{cryst}=\sum|F_{obs}-F_{calc}|/\sum|F_{obs}|$ where F_{obs} and F_{calc} are the observed and calculated structure factor amplitudes, respectively. R_{cryst} and R_{free} were calculated using the working and test sets, respectively.

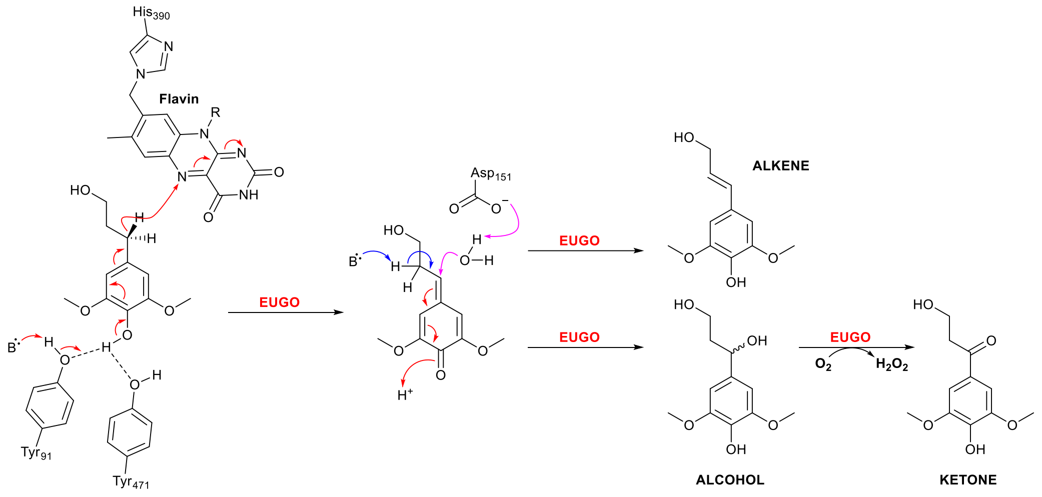

Figure S1. Mechanistic proposal for the oxidation of dihydrosinapyl alcohol by EUGO. Flavin acts as a hydride acceptor for dihydrosinapyl alcohol, generating a reactive quinone methide intermediate. The intermediate is quenched either by deprotonation of the adjacent carbon to form an alkene (blue arrows), or base-catalysed hydroxylation by a water molecule (pink arrows).

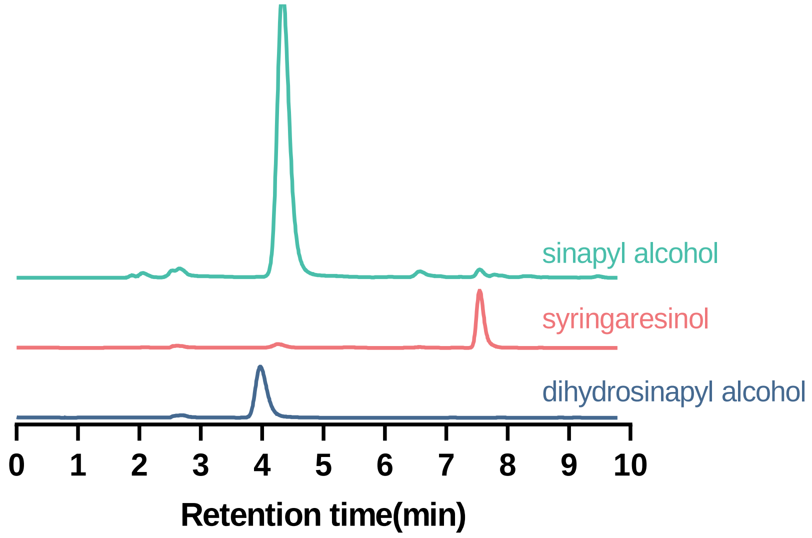

Figure S2. HPLC analysis of relevant compounds. Dihydrosinapyl alcohol, sinapyl alcohol, and syringaresinol are shown in blue, green and red, respectively.

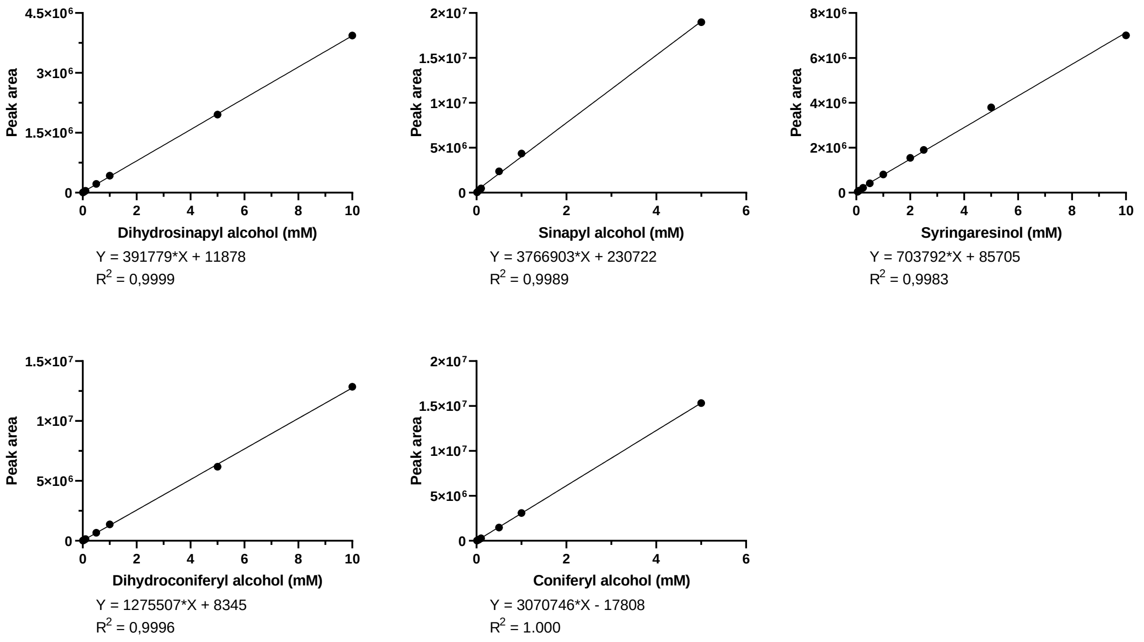

Figure S3. HPLC calibration curves of dihydroconiferyl alcohol, coniferyl alcohol, dihydrosinapyl alcohol, sinapyl alcohol and syringaresinol.

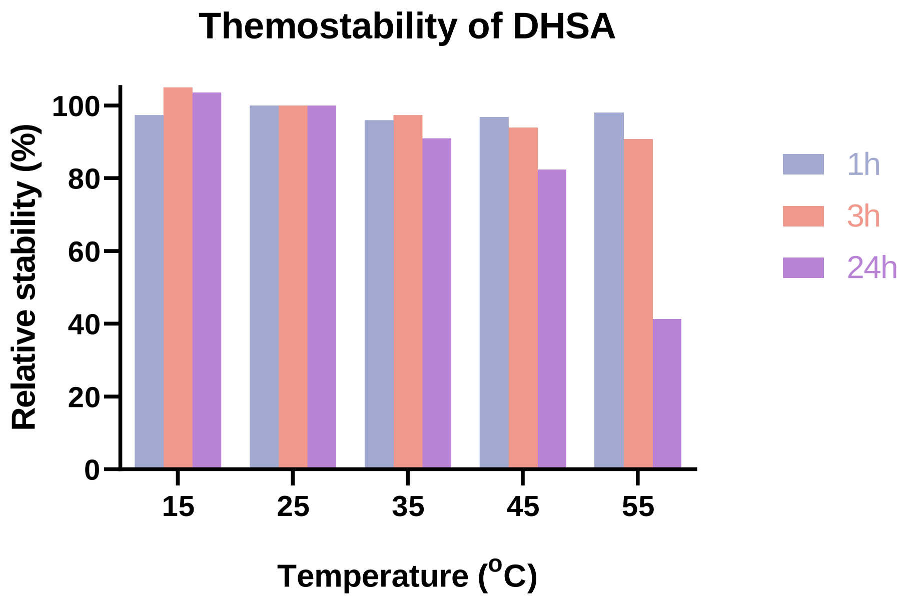

Figure S4. Temperature effect on stability of dihydrosinapyl alcohol. Dihydroconiferyl alcohol (5 mM) was incubated in KPi buffer (50 mM, pH 7.5) containing DMSO (10% v/v) at the indicated temperatures, and the mixture was assayed by HPLC.

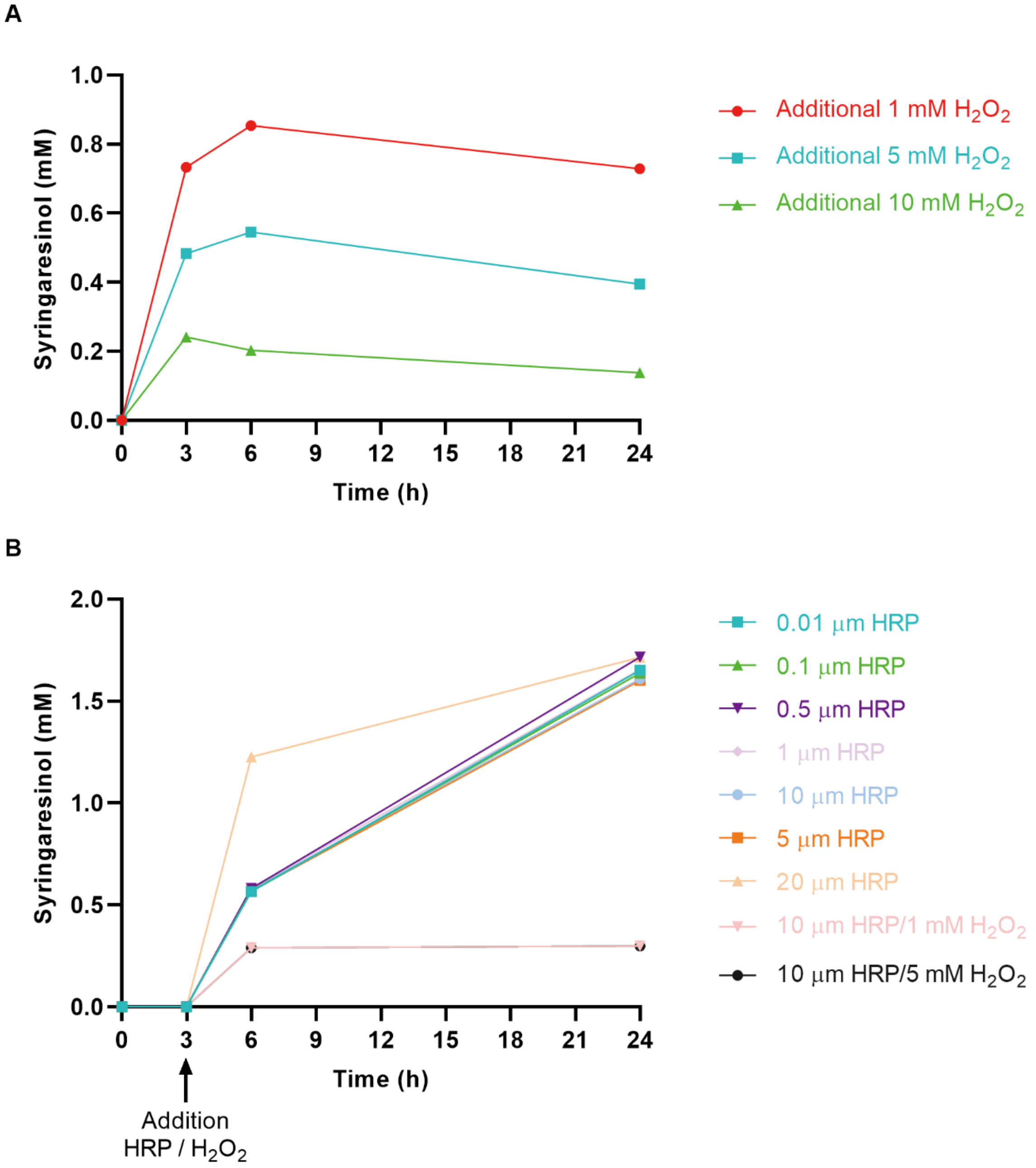

Figure S5. Optimization of the one-pot conversion of dihydrosinapyl alcohol to syringaresinol by an oxidase-peroxidase cascade reaction. A) Effect of additional hydrogen peroxide on the formation of syringaresinol from dihydrosinapyl alcohol by an oxidase-HRP cascade reaction. Using dihydrosinapyl alcohol (5 mM), oxidase (10 µM), and HRP (10 µM), the reactions with a gradient addition of hydrogen peroxide were performed in KPi buffer (50 mM, pH 7.5) at 37 °C, 150 rpm. B) Effects of delayed addition of HRP and hydrogen peroxide on the formation of syringaresinol by an oxidase-HRP cascade reaction. The reactions were initialized with dihydrosinapyl alcohol (5 mM) and oxidase (10 µM), and then HRP with a gradient concentration and HRP (10 µM) combined with hydrogen peroxide at 5 mM and 10 mM were added after 3 hours.

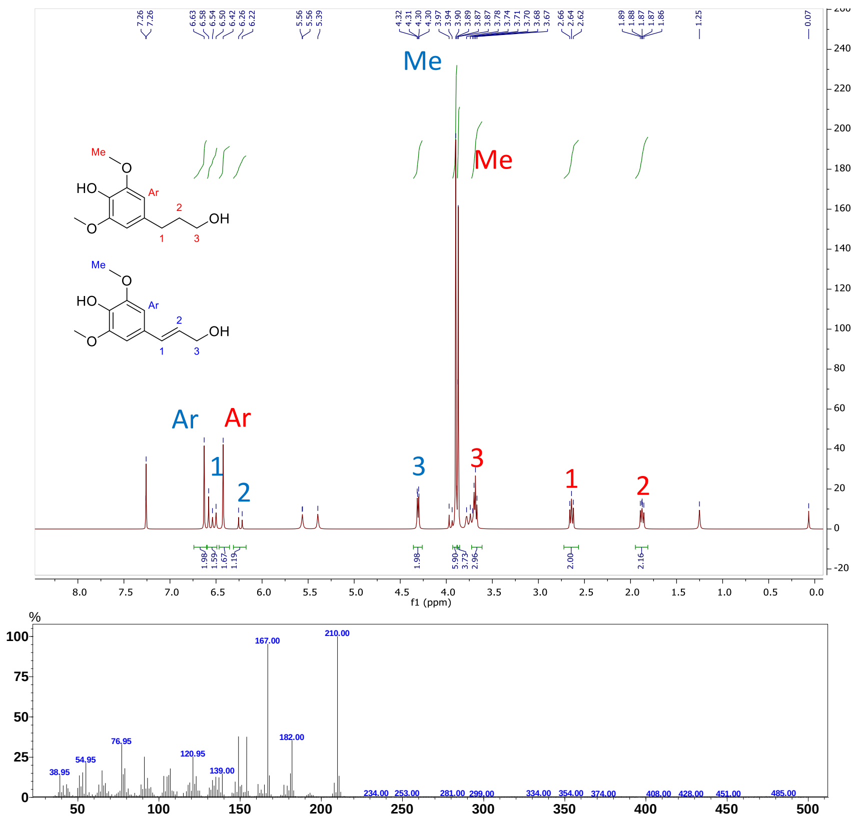

Figure S6. Analysis of product mixture isolated from 20 mg conversion of dihydrosinapyl alcohol using EUGO10X. A) ^{1}H NMR spectrum of product mixture of sinapyl alcohol and dihydrosinapyl alcohol. B) GC-EI-MS mass spectrum of the sinapyl alcohol reaction product. The NMR peaks formed which are distinct from those of dihydrosinapyl alcohol align well with literature values for sinapyl alcohol.[1]

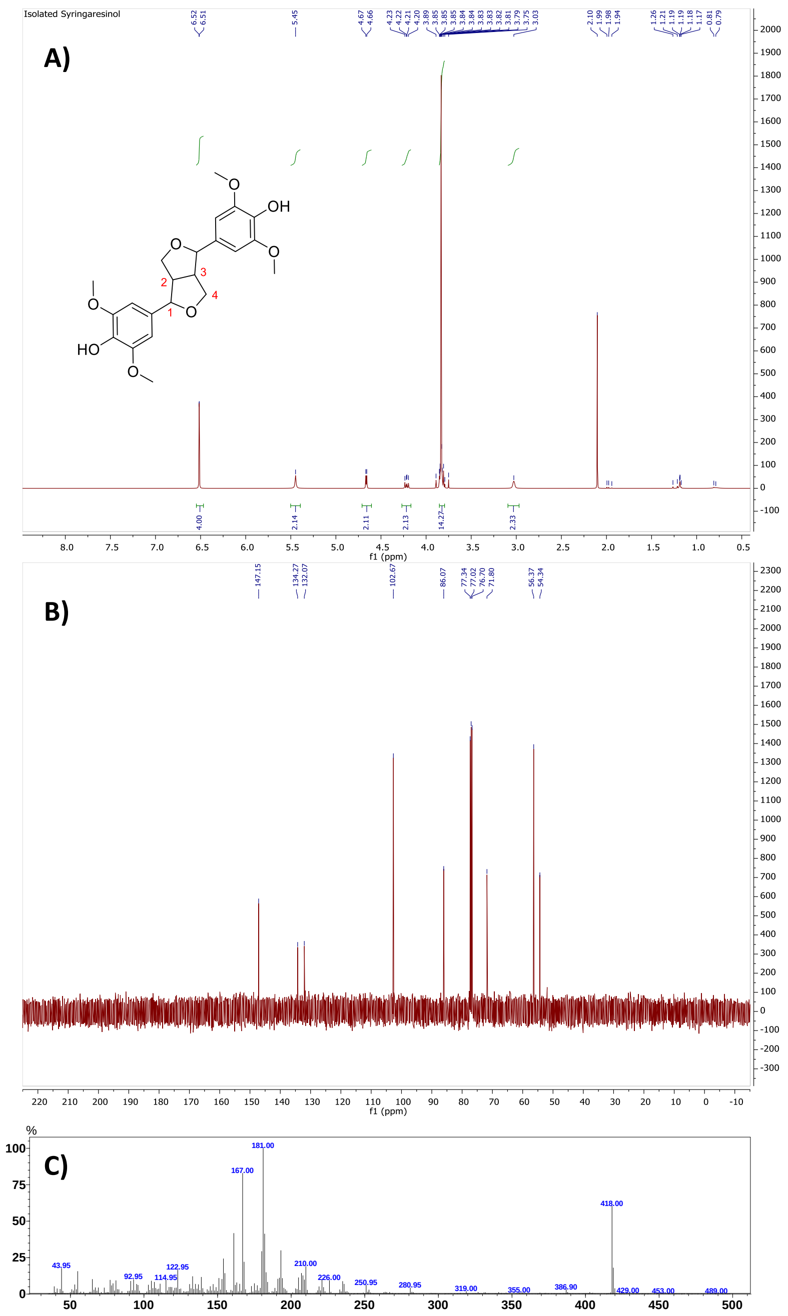

Figure S7. Analytical data of syringaresinol product isolated from 20 mg conversion of dihydrosinapyl alcohol using EUGO10X and HRP. A) ^{1}H NMR spectrum. B) ^{13}C NMR spectrum. C) GC-EI-MS mass spectrum of the syringaresinol product.

^{1}H NMR (400 MHz, $CDCl_3$) δ_H 6.51 (s, 4H, aromatic protons), 5.45 (s, 2H, -OH), 4.67 (d, J = 3.9 Hz, 2H, H-1), 4.22 (dd, 2H, J = 7.4, 6.5 Hz, H-4), 3.86 – 3.80 (m, 14H, H-4 & -OMe), 3.08 – 3.00 (m, 2H, H-2).

^{13}C NMR (101 MHz, $CDCl_3$) δ_C 147.2, 134.3, 132.1, 102.7, 86.1, 71.8, 56.4, 54.3.

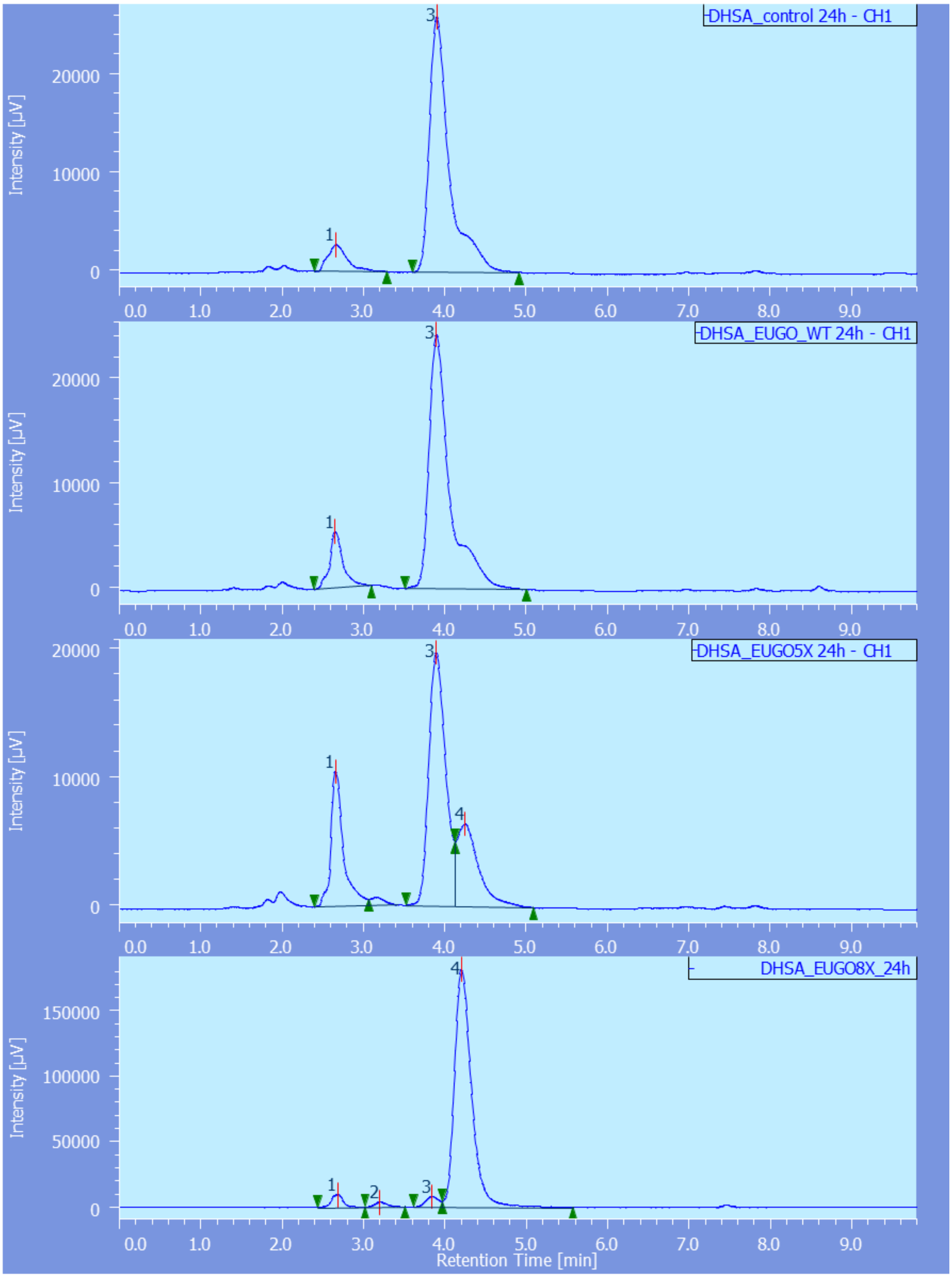

Figure S8. HPLC analysis of the conversion of dihydrosinapyl alcohol by EUGO variants at different stages of engineering. In all chromatograms, peaks 1, 2, 3 and 4 hydroxylated DHSA (Rt = 2.7 min), DHSA ketone (Rt = 3.2 min), DHSA (Rt = 4.0 min) and sinapyl alcohol (Rt = 4.3 min), respectively. The first chromatogram shows the elution of the reference substrate dihydrosinapyl alcohol. The chromatograms of the conversions show depletion of dihydrosinapyl alcohol to form sinapyl alcohol and by-products.

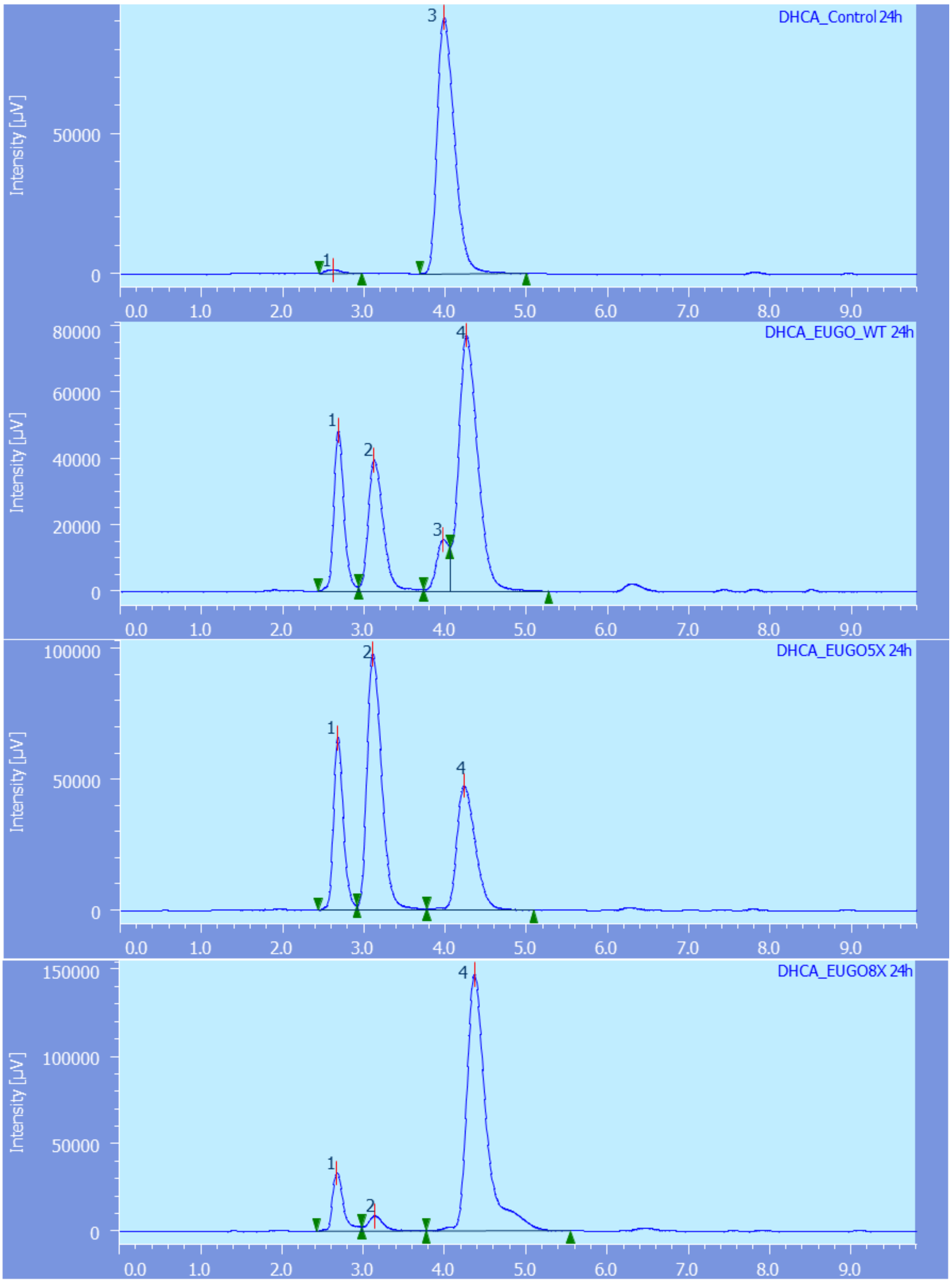

Figure S9. HPLC analysis of the conversion of dihydroconiferyl alcohol by EUGO variants at different stages of engineering. In all chromatograms, peak 1, 2, 3, 4 represent hydroxylated DHCA (Rt = 2.7 min), DHCA ketone (Rt = 3.2 min), DHCA (Rt = 4.0 min) and coniferyl alcohol (Rt = 4.3 min), respectively. The first chromatogram shows the elution of the reference substrate dihydroconiferyl alcohol. The chromatograms of the conversions show depletion of dihydroconiferyl alcohol to form coniferyl alcohol and by-products.

Protein Sequences

EUGO

MTRTLPPGVSDERFDAALQRFRDVVGDKWVLSTADELEAFRDPYPVGAAEANLPSAVVSP
ESTEQVQDIVRIANEYGIPLSPVSTGKNNGYGGAAPRLSGSVIVKTGERMNRILEVNEKY
GYALLEPGVTYFDLYEYLQSHDSGLMLDCPDLGWGSVVGNTLDRGVGYTPYGDHFMWQTG
LEVVLPQGEVMRTGMGALPGSDAWQLFPYGFGPFPDGMFTQSNLGIVTKMGIALMQRPPA
SQSFLITFDKEEDLEQIVDIMLPLRINMAPLQNVPVLRNIFMDAAAVSKRTEWFDGDGPM
PAEAIERMKKDLDLGFWNFYGTLYGPPPLIEMYYGMIKEAFGKIPGARFFTHEERDDRGG
HVLQDRHKINNGIPSLDELQLLDWVPNGGHIGFSPVSAPDGREAMKQFEMVRNRANEYNK
DYAAQFIIGLREMHHVCLFIYDTAIPEAREEILQMTKVLVREAAEAGYGEYRTHNALMDD
VMATFNWGDGALLKFHEKIKDALDPNGIIAPGKSGIWSQRFRGQNL

EUGO5X

MTRTLPPGVSDERFDAALQRFRDVVGDKWVLSTADELEAFRDPYPVGAAEANLPSAVVSP
ESTEQVQDIVRIANEYGIPLHPVSTGKNNGYGGAAPRLSGSVIVKTGERMNRILEVNEKY
GYALLEPGVTYFDLYEYLQSHDSGLMLDCPDLGWGSVVGNTLDRGVGYTPYGDHFMWQTG
LEVVLPQGEVMRTGMGALPGSDAWQLFPYGFGPFPDGMFTQSNLGIVTKMGIALMQRPPA
SQSFLITFDKEEDLEQIVDIMLPLRINMAPLQNVPVLRNIFMDAAAVSKRTEWFDGDGPM
PAEAIERMKKDLDLGFWNFYGTLYGPPPLIEMYYGMIKEAFGKIPGARFFTHEERDDRGG
HVLQDRHKINNGIPSLDELQLLDWVPNGGHIGFSPVSAPDGREAMKQFEMVRNRANEYNK
DYMAQFIIGLREMYHVCLFIYDTADPEAREEILQMTKVLVREAAEAGYGEYRTHNALMDD
VMATFNWGDGALLKFHEKIKDALDPNGIIAPGKSGIWPQRFRGQNL

EUGO8X

MTRTLPPGVSDERFDAALQRFRDVVGDKWVLSTADELEAFRDPYPVGAAEANLPSAVVSP
ESTEQVQDIVRIANEYGIPLHPVSTGKNNGYGGAAPRLSGSVIVKTGERMNRILEVNEKY
GYALLEPGVTYFDLYEYLQSHDSGLMLDCPDLGWGSVVGNTLDRGVGYTPYGDHFMWQTG
LEVVLPQGEVMRTGMGALPGSDAWQLFPYGFGPFPDGMFTQSNLGIVTKMGIALMQRPPA
SQSFLITFDKEEDLEQIVDIMLPLRINMAPLQNVPVLRNIFMDAAAVSKRTEWFDGDGPM
PAEAIERMKKDLDLGFWNFYGTLYGPPPLIEMYYGMIKEAFGKIPGARFFTHEERDDRGG
HVLQDRHKINNGIPSLDELQQLDWVPNGGHIGFVPVSAPDGREAMKQFEMVRNRANEYNK
DYMAQFVIGLREMYHVCLFIYDTADPEAREEILQMTKVLVREAAEAGYGEYRTHNALMDD
VMATFNWGDGALLKFHEKIKDALDPNGIIAPGKSGIWPQRFRGQNL

EUGO10X

MTRTLPPGVSDERFDAALQRFRDVVGDKWVLSTADELEAFRDPYPVGAAEANLPSAVVSP
ESTEQVQDIVRIANEYGIPLHPVSTGKNNGYGGAAPRLSGSVIVKTGERMNRILEVNEKY
GYALLEPGVTYFDLYEYLQSHDSGLMLDCPELGWGSVVGNTLDRGVGYTPYGDHFMWQTG
LEVVLPQGEVMRTGMGALPGSDAWQLFPYGFGPFPDGMFTQSNLGIVTKMGIALMQRPPA
SQSFLITFDKEEDLEQIVDIMLPLRINMAPLQNVPVLRNIFMDAAAVSKRTEWFDGDGPM
PAEAIERMKKDLDLGFWNFYGTLYGPPPLIEMYYGMIKEAFGKIPGARFFTHEERDDRGG
HVLQDRHKINNGIPSLDELQQLDWVPNGGHIGFVPVSAPDGREAMKQFEMVRNRANEYNK
DYMASFVIGLREMYHVCLFIYDTADPEAREEILQMTKVLVREAAEAGYGEYRTHNALMDD
VMATFNWGDGALLKFHEKIKDALDPNGIIAPGKSGIWPQRFRGQNL

BIBLIOGRAPHY

1. Zhao, C.; Hu, Z.; Shi, L.; Wang, C.; Yue, F.; Li, S.; Zhang, H.; Lu, F., Profiling of the formation of lignin-derived monomers and dimers from Eucalyptus alkali lignin. *Green Chem.* **2020,** *22* (21), 7366-7375.
